# Emergency penetrating keratoplasty in corneal perforations

**Published:** 2018

**Authors:** Alina-Cristina Stamate, Călin Petru Tătaru, Mihail Zemba

**Affiliations:** * Department of Ophthalmology, “Carol Davila” University of Medicine and Pharmacy, Bucharest, Romania; ** Arena Med Clinic, Bucharest, Romania; *** Clinical Hospital of Ophthalmologic Emergencies, Bucharest, Romania; **** Department of Ophthalmology, “Dr. Carol Davila” Central Military Emergency University Hospital, Bucharest, Romania

**Keywords:** corneal perforations, emergency, therapeutic penetrating keratoplasty

## Abstract

Corneal perforations represent an ophthalmological emergency due to their devastating consequences. Emergency treatment is mandatory to try to restore the anatomical integrity of the globe, to salvage useful vision as much as possible and to reduce the possible complications to a minimum. The underlying conditions or disorders responsible for corneal ulcerations, and subsequently for corneal perforations are numerous, and can be either isolated or superimposed. Emergency penetrating keratoplasty is a difficult surgical procedure that is associated with various complications, which can jeopardize the outcome of the eye.

## Introduction

Corneal perforations represent an ophthalmological emergency due to their devastating consequences. They are an important cause of ocular morbidity, leading to a decrease in vision and, in the worst-case scenario, even to blindness and loss of the eye. 

Emergency treatment is mandatory to try to restore the anatomical integrity of the globe, to salvage useful vision as much as possible and to reduce the possible complications to a minimum.

Such cases should be prevented, but, because of a late addressability to the doctor, delayed diagnosis, a low compliance to treatment, reduced availability to adequate drugs and inefficient response to therapy, these cases are still encountered, especially in developing countries [**1**].

In these hazardous situations, emergency penetrating keratoplasty plays an important role, both tectonically, in attempting to preserve the structure of the globe and also in some cases, in removing the infectious or inflammatory source.

Although in the last decade, penetrating keratoplasty has seen a serious decline, according to Park et al., and has been replaced by various types of lamellar keratoplasty [**2**], it is still the mainstay of care in central or large corneal perforations.

Lack of chances of visual recovery or reduced disponibility of corneal tissue, has called for desperate solutions in desperate situations, in the manner that various centers resorted to inventive methods to keep the eye in its place, such as using cryopreserved [**3**] or glycerol-preserved donor corneas [**4**], that have a long-term storage possibility, or even scleral autoplasty [**5**,**6**], autogenous periosteal graft from the anterior tibial crest [**7**], and multilayered Gore-Tex patches [**8**].

**Etiology**

The underlying conditions or disorders responsible for corneal ulcerations, and subsequently for corneal perforations are numerous, and can be either isolated or superimposed (**Table 1**).

**Table 1:** Conditions leading to corneal perforation

Infectious
bacterial, viral, fungal
Inflammatory
collagen vascular disease, Wegener’s granulomatosis, acne rosacea, atopic disease, Mooren’s ulcer
Trauma
penetrating, chemical, thermal
Surgical
cataract extraction, LASIK, PRK, pterygium excision with mitomycin-C, glaucoma filtering/shunt surgery
Xerosis
idiopathic, Sjӧgren’s syndrome, Stevens-Johnson syndrome, ocular cicatricial pemphigoid, vitamin A deficiency
Exposure
seventh nerve palsy, thyroid-related ophthalmopathy, ectropion, floppy eyelid syndrome
Neurotrophic
postviral, tumor, trauma, postsurgical
Degeneration/ectasia
Terrien’s marginal degeneration, keratoconus, keratoglobus, pellucid marginal degeneration
Toxic/keratolytic
topical NSAIDs, topical corticosteroids, topical antibiotics, silicone oil
Adapted from *Cornea. Surgery of the Cornea and Conjunctiva. Volume two* (p. 1572), by J.H. Krachmer, M.J. Mannis, E.J. Holland, 2011, St. Louis: Mosby Elsevier Inc. Copyright 2011 by Elsevier Inc.

The most common causes of corneal perforations are infections, inflammatory disorders, or trauma [**1**], with an oscillating predominance of non-infectious conditions in several studies [**9**-**12**] or infections as a leading cause in other studies [**13**].

In a descending order, the most common infections are bacterial, followed by viral infections and then fungal infections, which are less common [**13**].

Usually, inflammatory conditions, such as collagen vascular diseases (i.e. rheumatoid arthritis [**11**]), Wegener’s granulomatosis, and Mooren’s ulcer are responsible for peripheral perforations, and occasionally can cause central ulceration, followed by perforation [**14**].

Trauma, another important cause, has a devastating impact on the cornea, initially through direct injury, and later on, through corneal melting and necrosis.

Other causes include neurotrophic keratopathy, exposure keratopathy or corneal degenerations and ectatic disorders, and even intensive use of topical antibiotics or topical corticosteroids and nonsteroidal anti-inflammatory drugs.

**Pathogenesis**

The decisive trigger involved in the pathogenesis of corneal perforation is the breakdown of the corneal epithelium. The compromise of this barrier allows various pathogens to gain access into the corneal stroma and initiate an inflammatory response.

However, there are a few microorganisms, such as *Corynebacterium diphtheriae, Haemophilus aegyptius, Neisseria gonorrhoeae, Neisseria meningitidis and Shigella and Listeria species*, which can penetrate an intact corneal epithelium [**1**].

Corneal damage leading to corneal ulceration occurs as a combination of direct microbial invasion and a release of collagenases, secondary to host chemotaxis of leukocytes [**14**].

Other mechanisms involve direct tissue destruction (i.e. trauma) or severe thinning of the cornea that may predispose to perforation in case of minor trauma (i.e. corneal ectatic disorders).

## Clinical presentation

The clinical presentation of a patient with corneal perforation can be quite variable, depending on whether the perforation occurs in a healthy eye, in which case the patient will notice the symptoms immediately, or in a previously diseased one, in which case the patient may not sense some of the symptoms. 

The main symptoms in a corneal perforation are sudden decrease in visual acuity, pain and increased tearing.

Pain may be caused by ocular surface disease or can be secondary to iris or ciliary spasm or hemorrhagic choroidal detachments and increased tearing is a result of the sudden loss of aqueous humor.

Patients at risk of developing a corneal perforation should be advised to wear protective sunglasses during the day and a plastic shield at night, and most importantly to seek urgent medical care when they notice changes in their condition.

The most common signs of corneal perforation are a shallow or flat anterior chamber, positive Seidel test, uveal tissue prolapse, and hypotony (**Table 2**).

**Table 2:** Clinical presentation of corneal perforations

Symptoms
pain
decreased visual acuity
increased tearing
Signs
shallow or flat anterior chamber
positive Seidel test
uveal tissue to the posterior cornea or frank prolapse
hypotony
*Adapted from Cornea. Surgery of the Cornea and Conjunctiva. Volume two (p. 1573), by J.H. Krachmer, M.J. Mannis, E.J. Holland, 2011, St. Louis: Mosby Elsevier Inc. Copyright 2011 by Elsevier Inc*.

The examination of a patient with possible corneal perforation should be conducted with precaution and with minimal manipulation of the globe.

A thorough medical and ophthalmic anamnesis will offer information concerning the etiology of the perforation and will help initiate the proper treatment.

**Indications for therapeutic penetrating keratoplasty**

Corneal perforations are an ophthalmological emergency and require immediate medical and surgical treatment.

Considering the continuous progress of the surgical technique and instruments, the success rate of corneal transplants has greatly improved. Despite all these, some surgeons are reluctant to perform penetrating keratoplasty in such eyes [**1**,**15**].

However, some studies have shown that the outcome is better when a more rapid surgical approach is attempted, in terms of earlier rehabilitation and shorter periods of hospitalization [**15**, **16**].

If the anterior chamber is flat, regardless of method, repair should be performed in the first 24-48 hours to minimize the potential complications.

The choice of the treatment can be influenced by various factors: size and location of the perforation, etiology and immune status of the patient [**1**,**10**].

If the corneal defect is small, tissue adhesives, such as cyanoacrylate glue, multilayered amniotic membrane transplantation, conjunctival flap or patch graft can be used, but in cases of failure of previous treatments or larger perforations, more than 3 mm in diameter [**1**], penetrating keratoplasty should be performed.

**Management of corneal perforations**

The purpose of this manuscript is not to describe the already well-established surgical technique of elective penetrating keratoplasty, but rather to emphasize the special precautions that have to be taken in an emergency penetrating keratoplasty, the preoperative preparation of the patient, the adjustments in the surgical approach and technique, and also the postoperative management.

It is important to keep in mind that elective penetrating keratoplasty is performed for optical reasons, to achieve an improved optical clarity or to prevent corneal thinning leading to corneal descemetocele or perforations, and is a scheduled surgery, whereas emergency penetrating keratoplasty is a therapeutic procedure meant to restore the structure of the eye and eradicate infection and inflammation. 

In the second setting, the optical purpose is postponed, and pursued later, through various surgical procedures, after stability of the eye is achieved, or totally ignored, in cases in which there is no potential for visual rehabilitation.

**Preoperative management**

As highlighted previously, a careful medical and ophthalmic history should be performed to discover the cause of the perforation or the presence of any associated systemic comorbidities.

If an infection is suspected, corneal scrapings for stains and culture should be obtained, initial broad-spectrum antimicrobial therapy initiated and later appropriate systemic and topical antibiotics, antivirals, or antifungals administered according to laboratory findings [**15**]. 

If the corneal perforation is presumed to be sterile, the preoperative antibiotic prophylaxis should be broad spectrum and nontoxic to the cornea (e.g. topical fourth-generation fluoroquinolone associated with a systemic fluoroquinolone) and in inflammatory diseases, intensive topical and systemic immunosuppressive therapy should be used [**12**].

In these cases, general anesthesia is usually preferred, taking into consideration the patient’s medical history, and alerting the anesthesiologist of the open globe.

**Surgical technique**

After anesthesia is performed, a Barraquer eyelid speculum is gently inserted, making sure not to exert pressure on the globe. Also, if possible, a Flieringa ring can be sutured in place to provide scleral support. 

The difficulty of therapeutic penetrating keratoplasty lies in the trephination of the host cornea due to loss of eye rigidity. The purpose of this stage of the surgery is to remove all necrotic or infected tissue, and if possible, also a 1 mm rim of healthy corneal tissue, in order to leave behind a stable and infection free recipient bed.

Viscoelastic can occasionally be used to recreate the anterior chamber before trephination. 

The excision of the host cornea can be done by marking the superficial cornea with the trephine and afterwards deepening the mark with a disposable blade. The cornea is excised through the trephination mark. In some selected cases, a suction trephine can be used, because the system exerts the pressure outwards, making sure no to apply direct pressure on the globe when using it, and avoiding damage to the iris and lens [**1**,**17**]. 

Once the corneal button is removed, the anterior chamber is inspected for peripheral anterior synechiae, posterior synechiae, and cataract. 

**Fig. 1:**
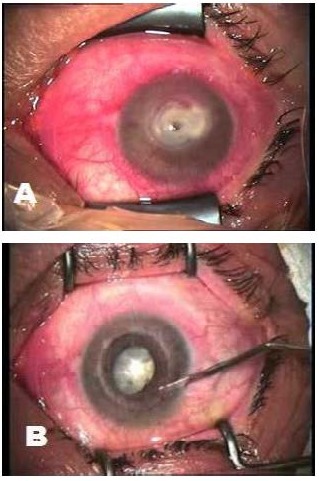
Corneal perforation. **A** aspect at the beginning of surgery. **B** anterior synechiolysis after performing emergency penetrating keratoplasty

Posterior and anterior synechiae should be gently lysed, peripheral iridectomies performed, and the anterior chamber irrigated to remove all necrotic or inflammatory debris (**Fig. 1**). 

**Fig. 2:**
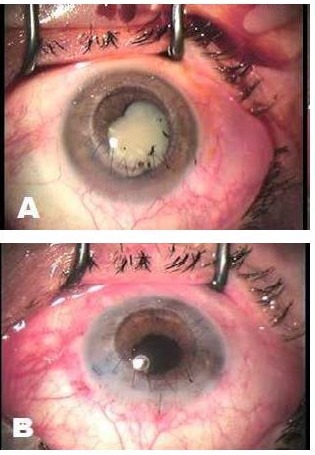
Emergency penetrating keratoplasty for corneal perforation. **A** visualization of cataract at the end of the surgery. **B** cataract extraction in the same patient in a second surgical intervention

In most cases, cataract is extracted in a following intervention, due to a high risk of expulsive hemorrhage, vitreous loss, and endophthalmitis (**Fig. 2**).

In long standing cases, encountered especially in developing countries, the iris prolapses into the corneal wound and it is difficult not to damage it during surgery, by causing severe bleeding and performing large surgical excisions [**1**].

**Postoperative management**

Postoperative management of therapeutic penetrating keratoplasty can be troublesome, but some targets should be established: 

- to eliminate infection and prevent reinfection, 

- to obtain reepithelialization of the cornea and healing of the wound,

- to control inflammation with corticosteroids (controversial in fungal infections),

- to monitor intraocular pressure [**14**].

Close follow-up of these patients is mandatory.

**Complications**

Considering that emergency keratoplasty is a high risk procedure, a multitude of complications are possible: persistent epithelial defects, recurrence of corneal melting, graft rejection, late graft failure, recurrence of infection, glaucoma, cataract, persistent anterior chamber leakage, ingrowth of corneal and conjunctival epithelium, inflammatory membranes, retinal or choroidal detachment, endophthalmitis, or phthisis bulbi [**9**,**11**,**15**,**18**].

**Outcomes and visual prognosis**

The clinical outcome of a surgery performed in emergent conditions may differ from that expected in an electively planned one.

So, in these cases, the visual prognosis is set aside as a second objective because it can be highly variable, being dependent on numerous factors: etiology of perforation, associated ocular diseases, severity of inflammation at the time of the surgery (that can lead to donor corneal melting, vascularization, and graft rejection) or the size of the graft.

In a recent study, Yokogawa et al. reported that the success rate of therapeutic penetrating keratoplasty for infectious keratitis is influenced by: microbial organisms virulence, predisposing factor, extensiveness of preexisting keratitis, associated ocular surface inflammation, initial medical treatment and surgical techniques [**10**].

Other studies that evaluated the outcome of corneal perforations, revealed that therapeutic penetrating keratoplasties for infectious conditions carry a better prognosis than those performed for immunologic conditions [**12**,**19**,**20**].

Regarding visual acuity, several studies presented their results. In a study by Jonas et al., best corrected visual acuity ranged from perception of light to 0.80 (median, 0.10), with 90% of the patients attaining an improvement of best visual acuity during the follow-up period [**9**], and in another study by Anshu et al., 20.2% of the patients achieved a best corrected visual acuity of 6/ 9 or greater [**21**].

One of the largest retrospective studies performed by Hossain et al. showed that 1330 emergency corneal grafts were performed on a period of 6 years in the UK, out of which 65.9% were for corneal perforations. Best corrected visual acuity of surviving grafts at 1 year was: 6/ 12 or better in 29.9%, 6/ 18 to 6/ 60 in 38.4%, counting fingers to light perception in 30% and no light perception in 1% of the cases, with worsening of vision in only 8.7% of the patients [**13**].

## Conclusions

Corneal perforations are ocular emergencies with devastating consequences. The main goal of the treatment is to maintain the anatomical structure of the globe and visual recovery is a secondary objective.

Therapeutic penetrating keratoplasty is an extremely difficult surgical procedure that is associated with various complications, which can jeopardize the outcome of the eye.

The success rate of emergency keratoplasty in corneal perforations reported by various studies reinforces the importance of eye banking in supplying corneal grafts in such conditions.

**Disclosures**

None of the authors has any financial or proprietary interests to disclose.
